# A novel tumor suppressor gene ECRG4 interacts directly with TMPRSS11A (ECRG1) to inhibit cancer cell growth in esophageal carcinoma

**DOI:** 10.1186/1471-2407-11-52

**Published:** 2011-02-03

**Authors:** Lin-wei Li, Yuan-yuan Li, Xiao-yan Li, Chun-peng Zhang, Yun Zhou, Shih-Hsin Lu

**Affiliations:** 1 Oncology Department, Henan Provincial People's Hospital, Zhengzhou 450003, P.R. China; 2 State Key Laboratory of Molecular Oncology and Department of Etiology and Carcinogenesis, Cancer Institute & Hospital, Chinese Academy of Medical Sciences & Peking Union Medical College, Beijing 100021, P.R. China

## Abstract

**Background:**

The esophageal carcinoma related gene 4 (ECRG4) was initially identified and cloned from human normal esophageal epithelium in our laboratory (GenBank accession no.AF325503). ECRG4 has been described as a novel tumor suppressor gene associated with prognosis in esophageal squamous cell carcinoma (ESCC).

**Methods:**

In this study, binding affinity assay in vitro and co-immunoprecipitation experiment in vivo were utilized to verify the physical interaction between ECRG4 and transmembrane protease, serine 11A (TMPRSS11A, also known as ECRG1, GenBank accession no. AF 071882). Then, p21 protein expression, cell cycle and cell proliferation regulations were examined after ECRG4 and ECRG1 co-transfection in ESCC cells.

**Results:**

We revealed for the first time that ECRG4 interacted directly with ECRG1 to inhibit cancer cell proliferation and induce cell cycle G1 phase block in ESCC. Binding affinity and co-immunoprecipitation assays demonstrated that ECRG4 interacted directly with ECRG1 in ESCC cells. Furthermore, the ECRG4 and ECRG1 co-expression remarkably upregulatd p21 protein level by Western blot (P < 0.001), induced cell cycle G1 phase block by flow cytometric analysis (P < 0.001) and suppressed cell proliferation by MTT and BrdU assay (both P < 0.01) in ESCC cells.

**Conclusions:**

ECRG4 interacts directly with ECRG1 to upregulate p21 protein expression, induce cell cycle G1 phase block and inhibit cancer cells proliferation in ESCC.

## Background

Esophageal carcinoma ranks 7^th ^and 6^th ^in terms of cancer incidence and mortality rate worldwide, respectively [1]. Moreover, nearly 50% of esophageal carcinoma cases in the world occurred in China [2]. Esophageal squamous cell carcinoma (ESCC), which is the most common histological subtype, accounts for ~90% of all esophageal cancers diagnosed in China each year. Despite advances in clinical comprehensive treatment, ESCC prognosis remains poor due to its diffuse and invasive nature. To date, the molecular pathogenesis of ESCC is still unclear [3,4]. At present, the focus of biology studies is transitioning from the cloning of novel genes to characterizing the function of the protein product. As a result, a major research effort has been directed at identifying the function of novel specific esophageal cancer related genes and elucidating the relevant molecular interactions of protein products which may play critical roles in ESCC.

The ECRG4 gene (GenBank accession no. AF 325503) was initially identified and cloned in our laboratory from human normal esophageal epithelium [5-7]. Either ECRG4 RNA or ECRG4 protein was an independent prognostic factor for ESCC, and the low expression of ECRG4 gene in patients with ESCC was associated with poor prognosis [8,9]. Furthermore, ECRG4 overexpression in ESCC cells inhibited tumor cells growth and invasion [9,10]. And recent studies showed that ECRG4 might be involved in the development of multi-tumors [11-13].

In the present study, we further explored the functional interaction between ECRG4 and transmembrane protease, serine 11A (TMPRSS11A, also known as ECRG1) to induce cell cycle G1 phase block and suppress cell growth in ESCC.

## Methods

### Construction of eukaryotic expression vector and transfection

The coding region of ECRG4 or ECRG1 cDNA was subcloned into constitutive mammalian expression vector pcDNA3.1 (Invitrogen). The cDNA was then fully sequenced to ensure that no mutation was introduced during the PCR amplification. The resulting plasmid construct was named pcDNA3.1-His-ECRG4 and pcDNA3.1-FLAG-ECRG1. The human esophageal squamous cell line EC9706 was established and studied by Han *et al *[14]. EC9706 cells were transfected with pcDNA3.1-His-ECRG4 or pcDNA3.1-FLAG-ECRG1 using lipofectamine™ 2000 (Invitrogen, CA), according to the manufacturer's protocol.

### Produce and purification of recombinant ECRG4 protein

The ECRG4 cDNA was excised from pGEM-T-ECRG4 and subcloned into the pET30a (+) plasmid, producing an inducible expression vector coding for His-tagged ECRG4 soluble protein. Subsequently, the recombinant plasmids were transformed into *Escherichia coli *BL21 (DE3) cells to produce N-terminal His-tagged soluble ECRG4 protein. Fusion protein expression in *Escherichia coli *BL21 cells was induced with 0.3 mM isopropyl-D-thiogalactopyranoside (IPTG), and the protein was purified by affinity chromatography with nickel-nitrilotriacetic acid (Ni-NTA) resin (Novagen), according to the manufacturer's protocol. The purified fusion protein was dialyzed in phosphate-buffered saline (PBS; 0.1 M sodium phosphate and 0.15 M sodium chloride [pH 7.4]) to remove the denaturant [9].

### Western blot analysis

Whole-cell lysates of EC9706 cells were prepared by incubating cells in RIPA buffer (1% NP-40; 0.5% sodium deoxycholate; 0.1% SDS; 50 mM Tris-HCl [pH 7.5]) containing protease inhibitors. Cell lysates were centrifuged at 10,000 g for 10 minutes at 4°C. The supernatant was collected, and the protein concentration was measured using the BCA™ Protein Assay Kit (Pierce). Proteins (40 ug) in cell lysates or culture media were separated by 10-15% SDS-polyacrylamide gel electrophoresis and transferred onto PVDF membrane. The membranes were blocked in TBST (0.2 M NaCl; 10 mM Tris pH7.4; 0.2% Tween20)/5% skim milk for 2 hours at room temperature and then incubated with primary antibodies in TBST/5% skim milk. The primary antibodies used for Western blot analysis were monoclonal mouse anti-His (1:4000), monoclonal mouse anti-FLAG (1:4000), polyclonal rabbit anti-p21 (1:4000) and monoclonal mouse anti-β-actin (1:4000). The membranes were then washed three times with TBST, followed by incubation with horseradish peroxidase-conjugated secondary antibody (1:4000) in TBST/5% skim milk. Bound antibody was visualized using ECL detection reagent.

### Cell proliferation assays

Cell growth and viability were evaluated by using MTT and BrdU assays, respectively. For MTT assay, the transfected cells were seeded into 96-well plates (1.5 × 10^3 ^cells/well). After culturing for various durations, cell proliferation was evaluated by thiazolyl blue tetrazolium bromide (MTT) assay, according to the manufacturer's protocol (Sigma-Aldrich Co., St. Louis, MO, USA). In brief, 10 μl MTT solution (5 mg/ml) was added to each well, then the cells were cultured for another 4 hours at 37°C, and 100 μl DMSO was added to each well and mix vigorously to solubilize colored crystals produced within the living cells. The absorbance at 570 nm was measured by using a multi-well scanning spectrophotometer Victor 3. For the BrdU assay, the transfected cells were seeded into 96-well plates (1 × 10^5 ^cells/well). After transfection for two days, the BrdU assay (BrdU cell proliferation ELISA, Roche) was carried out according to the manufacture's instructions.

### Flow cytometric analysis of cell cycle

The transfected cells were seeded at a density of 10^6 ^cells/100-mm dish in RPMI-1640 medium with 10% FBS for 48 hours. Then cells were washed with ice-cold PBS, harvested and fixed in 70% ethanol for 30 minutes. Cells were treated with RNase A and stained with 25 μg/ml propidium iodide (PI). Samples were analyzed using a FACScan flow cytometer (Becton Dickinson), according to the manufacturer's protocol. Experiments were performed three times in triplicate.

### Binding affinity assay

Recombinant purified His-ECRG4 protein was coated on 96-well microtiter plates (5 μg per well) followed by bovine serum albumin blocking. The whole-cell lysates of EC9706 cells with FLAG-ECRG1 transfection was then added to the wells and incubated for 2 h. After washing, anti-FLAG or anti- Miz-1 antibody was added to the wells and incubated for 30 min at 37°C. Horseradish peroxidase-conjugated secondary antibody (Santa Cruz, CA) was added to the wells and incubated for 20 min at 37°C. After incubation, the substrate o-phenylenediamine dihydrochloride was added to the wells, and the colored reaction product was quantified using a microplate reader at 490 nm [15].

### Co-immunoprecipitation

Immunoprecipitation and Western blot analysis was performed according to the standard protocol (Sigma). EC9706 cells were co-transfected with pcDNA3.1-His-ECRG4 (10 μg) and pcDNA3.1-FLAG-ECRG1 (3.3 μg) or with control vectors pcDNA3.1 (10 μg) in 10 cm dishes using Lipofectamine™2000 (Invitrogen, CA). Two days after transfection, cells were solubilized with 1 ml of lysis buffer (50 mM Tris-HCl [pH7.5], 150 mM NaCl, 1% Nonidet P40, 0.5% sodium deoxycholate) (Roche) on ice for 30 minutes. Insoluble materials were removed by centrifugation for 20s at 12,000 g at 4°C. The supernatant was collected and the protein concentration was measured by Bradford method to be adjusted to a final concentration of 1 mg/ml. The supernatant was precleared with Protein G (Roche) for 3 h at 4°C. Then 500 μl of lysate was incubated with anti-His or anti-FLAG antibody coupled to protein G-Sepharose beads overnight at 4°C with gentle rotation. The beads were washed with wash buffer (Wash 1: 50 mM Tris-HCl (pH7.5), 150 mM NaCl, 1% NP40, 0.5% sodium deoxycholate; Wash 2: 50 mM Tris-HCl [pH7.5], 500 mM NaCl, 0.1% NP40, 0.05% sodium deoxycholate; Wash 3: 10 mM Tris-HCl [pH7.5], 0.1% NP40, 0.05% sodium deoxycholate). The immunocomplex retained on the beads were eluted in 2× Laemmli buffer (20% glycerol, 2%SDS, 250 mM Tris pH6.8, 10% β-mercaptoethanol and 0.1% bromophenol blue), boiled and microcentrifuged. Supernatant proteins were subjected to 12% SDS-polyacrylamide gel electrophoresis, and immunoblot analysis for anti-FLAG or anti-His were performed as described above.

### Statistical analysis

All statistical analysis was performed with the SPSS statistical program (version 13.0). *P *< 0.05 was considered statistically significant.

## Results

### ECRG4 bind to ECRG1 *in vitro*

**Figure 1:**
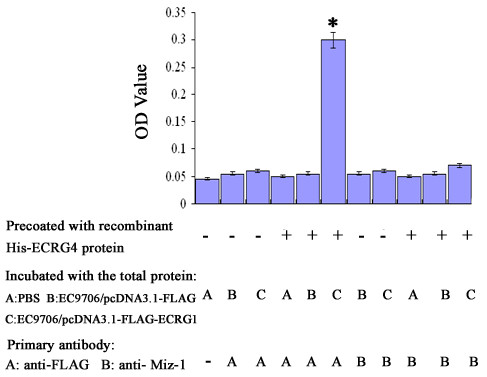
**The interaction between ECRG4 and ECRG1 was examined by binding affinity assay *in vitro***. FLAG-ECRG1 from EC9706/pcDNA3.1-FLAG-ECRG1 cells was bound to recombinant His-ECRG4 protein which had been pre-coated on the plate. The absorbance values of the wells (column 6) were significantly higher than those of controls (*P *< 0.001). And no detectable binding of Miz-1, an interaction partner of ECRG1, to His-ECRG4 was observed. *, *P *< 0.001, compared with EC9706/pcDNA3.1-FLAG cells.

Our previous results demonstrated that both ECRG4 and ECRG1 inhibited cell proliferation and induced cell cycle G1 phase block in ESCC. Furthermore, online bioinformatics analysis database Swiss-Prot predicted that ECRG4 could potentially bind to ECRG1 by protein-protein interaction. Therefore, we speculated that the tumor suppressor genes ECRG4 and ECRG1 might interact physically in ESCC. To explore this possibility, binding affinity assay was performed to test the interaction between ECRG4 and ECRG1 proteins. Recombinant His-ECRG4 protein was precoated into the wells of 96-well plate and incubated with the total protein from EC9706/pcDNA3.1-FLAG-ECRG1 or EC9706/pcDNA3.1-FLAG cells. Bound protein was detected by anti-FLAG antibody. As the control for background protein binding, bovine serum albumin was coated into the wells of 96-well plate and incubated with PBS. The FLAG-ECRG1 protein exhibited binding to recombinant His-ECRG4 protein (Figure 1). And no detectable binding of Miz-1, an interaction partner of ECRG1, to His-ECRG4 was observed.

### ECRG4 interacted directly with ECRG1 *in vivo*

**Figure 2:**
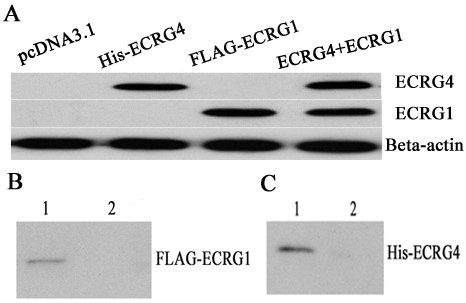
**The interaction between ECRG4 and ECRG1 was verified by co-immunoprecipitation assay *in vivo***. (A) Detection of ECRG4 and ECRG1 protein expression in transfected cells (pcDNA3.1, pcDNA3.1-His-ECRG4, pcDNA3.1-FLAG-ECRG1 and pcDNA3.1-His-ECRG4+FLAG-ECRG1) by Western blot. FLAG-ECRG1 or His-ECRG4 protein was detectable in EC9706/pcDNA3.1-FLAG-ECRG1 or EC9706/pcDNA3.1-His-ECRG4 cells, respectively, and both in EC9706/pcDNA3.1-His-ECRG4+pcDNA3.1-FLAG-ECRG1 cells. (B) EC9706 cells, transiently transfected with FLAG-ECRG1 and His-ECRG4, were immunoprecipitated by anti-FLAG antibody and detected by anti-His antibody. As negative control, pcDNA3.1-FLAG empty vector replaced FLAG-ECRG1. Protein lysates of EC9706/FLAG-ECRG1+His-ECRG4 (Lane 1) and EC9706/FLAG+ His-ECRG4 (Lane 2) were immunoprecipitated by anti-FLAG antibody and visualized by anti-His antibody. The rabbit IgG antibody was used as negative control, and it showed no non-specific binding of ECRG1 with IgG. (C) EC9706 cells, transiently transfected with FLAG-ECRG1 and His-ECRG4, were immunoprecipitated by anti-His antibody and detected by anti-FLAG antibody. As negative control, pcDNA3.1-His empty vector replaced His-ECRG4. Protein lysates of EC9706/FLAG-ECRG1+His-ECRG4 (Lane 1) and EC9706/FLAG-ECRG1+His (Lane 2) were immunoprecipitated by anti-His antibody and visualized by anti-FLAG antibody. The rabbit IgG antibody was used as negative control, and it showed no non-specific binding of ECRG4 with IgG.

To investigate whether the interaction between ECRG4 and ECRG1 in ESCC cells occurred *in vivo*, the co-immunoprecipitation assay was utilized to verify the possibility. And in order to get a better understanding of the physical association of ECRG4 and ECRG1, the ECRG1 and ECRG4 null ESCC cell line EC9706 was utilized to be transfected with ECRG1 and ECRG4 gene, respectively. The FLAG-ECRG1 or His-ECRG4 protein was detected in EC9706/pcDNA3.1-FLAG-ECRG1, EC9706/pcDNA3.1-His-ECRG4, and EC9706/pcDNA3.1-His-ECRG4+FLAG-ECRG1 cells, respectively (Figure 2A). FLAG tagged ECRG1 protein was transiently co-expressed in EC9706 cells together with His-ECRG4. As negative control, pcDNA3.1-FLAG empty vector replaced pcDNA3.1-FLAG-ECRG1 in EC9706 cells. The cell lysates with high protein expression of His-ECRG4 and FLAG-ECRG1 detected by immunoblotting were then immunoprecipitated with anti-FLAG monoclonal antibody. Immunoprecipitated proteins were separated by SDS-PAGE and immunoblotted with anti-His monoclonal antibody. The results showed that His-ECRG4 was present in the immunoprecipitates from cells expressing both FLAG-ECRG1 and His-ECRG4 proteins, but not in the control group (Figure 2B). In the inverse experiment, pcDNA3.1-His empty vector replaced pcDNA3.1-His-ECRG4 as negative control. The protein lysates were immunoprecipitated with anti-His antibody and immunoblotted with anti-FLAG antibody. Consistently, the results demonstrated that the immunoprecipitates contained FLAG-ECRG1 protein when the cells were co-transfected with pcDNA3.1-His-ECRG4 and pcDNA3.1-FLAG-ECRG1 genes, but not in the control group (Figure 2C). Altogether, the co-immunoprecipitation assays *in vivo *supported the interaction between ECRG4 and ECRG1 proteins in ESCC cells.

### ECRG4 and ECRG1 co-expression increased p21 expression

**Figure 3:**
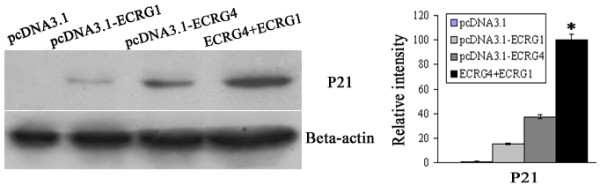
**Effect of ECRG4 and ECRG1 co-expression on p21 protein level. Representative photo (left) and statistic plot (right) of relative protein expression ratios in EC9706/pcDNA3.1, EC9706/pcDNA3.1-ECRG1, EC9706/pcDNA3.1-ECRG4 and EC9706/pcDNA3.1-ECRG4+ECRG1 cells**. Analysis of cell's total proteins by Western blot showed that p21 protein expression was significantly increased in EC9706/pcDNA3.1-ECRG4+ECRG1 cells compared with in EC9706/pcDNA3.1 cells (*P *< 0.001). *, *P *< 0.001, compared with EC9706/pcDNA3.1 cells.

The interaction between ECRG4 and ECRG1 indicated that the two proteins might be involved in same physiological process. So we examined the effect of ECRG4 and ECRG1 co-transfection in EC9706 cells on cell cycle G1 phase regulation gene p21 expression. The EC9706 cells were transfected with ECRG1 and ECRG4 genes, alone or combined, and p21 expression was analyzed by Western blot assay. The result showed that either ECRG4 or ECRG1 transfection could upregulat the p21 expression level. Furthermore, the co-transfection of ECRG4 and ECRG1 remarkably reinforced the upregulation effect in EC9706 cells (*P *< 0.001) (Figure 3). The results suggested that the ECRG4 and ECRG1 co-expression enhanced p21 upregulation in ESCC cells.

### ECRG4 and ECRG1 co-expression reinforced cell cycle G1 phase block

Cell cycle examination was carried out by flow cytometric analysis in an attempt to explore the effect of p21 upregulatin induced by ECRG4 and ECRG1 co-expression. The result suggested that either ECRG4 or ECRG1 expression could arrest ESCC cells at the G1/S checkpoint and delay cell cycle into S phase. Furthermore, the ECRG4 and ECRG1 co-expression reinforced the cell cycle G1 phase block effect (*P *< 0.001) (Table 1). Consequently, ECRG4 and ECRG1 co-expression slowed down cell cycle progression and enhanced cell cycle G1 phase block in ESCC cells.

**Table 1:** ECRG4 and ECRG1 induced cell cycle G1 phase block

Group	G1	S	G2/M
EC9706/pcDNA3.1	60.1 ± 2.11	24.5 ± 1.53	15.4 ± 1.67
EC9706/pcDNA3.1-ECRG4	70.6 ± 1.62	15.7 ± 1.35	13.7 ± 0.87
EC9706/pcDNA3.1-ECRG1	66.9 ± 1.58	19.6 ± 1.41	13.5 ± 1.03
EC9706/pcDNA3.1-ECRG4+ECRG1^#^	75.8 ± 1.82	11.3 ± 1.37	12.9 ±1.16

^# ^*P *< 0.001, compared with EC9706/pcDNA3.1.

### ECRG4 and ECRG1 co-expression inhibited tumor cell growth

**Figure 4:**
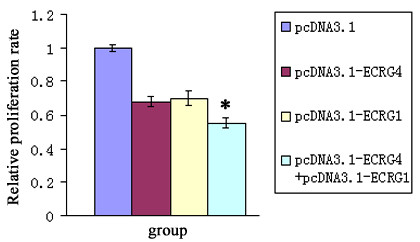
**Effect of ECRG4 and ECRG1 co-expression on cell proliferation**. EC9706/pcDNA3.1-ECRG4+ECRG1 cells proliferated significantly more slowly than EC9706/pcDNA3.1 cells as determined by the BrdU assay (*P *< 0.01). *, *P *< 0.01, compared with EC9706/pcDNA3.1 cells. Error bars represent standard deviation from mean value.

**Figure 5:**
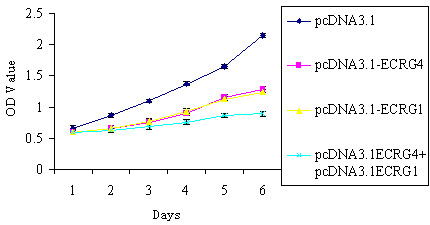
**Effect of ECRG4 and ECRG1 co-expression on cell growth curve**. Cell growth curves demonstrated that EC9706/pcDNA3.1-ECRG4+ECRG1 grew significantly more slowly than EC9706/pcDNA3.1 cells by MTT assay (*P *< 0.01). *, *P *< 0.01, compared with EC9706/pcDNA3.1 cells. Error bars represent standard deviation from mean value.

To further evaluate the inhibitory effect of ECRG4 and ECRG1 co-expression on cancer cell growth, the MTT and BrdU assays were utilized to examine cell viability and proliferation. The proliferation of EC9706 cells with either ECRG4 or ECRG1 transfection were inhibited compared with the control cells. Furthermore, the ECRG4 and ECRG1 co-transfection significantly reinforced the cell proliferation inhibition effect, as assessed by the BrdU assay (*P *< 0.01) (Figure 4). Moreover, cell growth curves also demonstrated that ECRG4 and ECRG1 co-transfection significantly slowed down cancer cells growth by MTT assay (*P *< 0.01) (Figure 5). The results indicated that ECRG4 and ECRG1 co-expression significantly enhanced the growth-suppressing effect in ESCC cells.

## Discussion and Conclusions

ESCC is a highly invasive and clinically challenging cancer in China. Until now, its molecular basis remains poorly understood. And ECRG4 gene is highly conserved among various species, suggesting an important role for ECRG4 in eukaryotic cells. However, its exactly biological function in carcinogenesis is still unclear. Our previous study demonstrated that ECRG4 is a novel tumor suppressor gene in ESCC. ECRG4 gene promoter hypermethylation accounted for decreased expression in ESCC, and the low expression of ECRG4 protein in patients with ESCC was associated with poor prognosis [7,9]. These findings were also supported by similar studies of other research groups [8,11,12]. Furthermore, restoration of ECRG4 expression in tumor cells inhibited cell growth and invasion [9,10,13]. And ECRG4 was also involved in cell differentiation and senescence [16-19].

ECRG1 (GenBank accession no. AF 071882) was also the candidate tumor suppressor gene in ESCC. The ECRG1 290 Arg/Gln and Gln/Gln genotypes were associated with increased risk for squamous cell carcinoma, compared with that of 290 Arg/Arg [20-22]. Our previous results demonstrated that overexpression of ECRG1 gene in ESCC cells inhibited tumor cells growth *in vitro *and *in vivo *[23,24]. Furthermore, ECRG1 could induce cell cycle G1 phase arrest and cell senescence through interaction with Miz-1 protein in ESCC cells [25-27]. These findings indicated that ECRG1 played an important role in controlling the gene expression involved in cell cycle G1 phase regulation and cell proliferation in ESCC.

Our data demonstrated that ECRG4 could also cause cell cycle G1 phase block by inducing p21 upregulation in ESCC cells [10]. And Bioinformatics analysis indicated that ECRG4 might be directly associated with ECRG1 by protein-protein interaction. In this study, binding affinity assay also demonstrated that ECRG4 could bind to ECRG1. Therefore, we reasoned that ECRG4 might interact with ECRG1 to co-regulate cell cycle and cell proliferation. As the binding affinity assay provided only potential interaction, we further performed co-immunoprecipitation assay *in vivo *to confirm the biological interaction between ECRG1 and ECRG4 in ESCC cells. In order to get a better understanding of the association of ECRG1 and ECRG4 on cell cycle and cell proliferation, as well as various physiological processes, the ECRG1 and ECRG4 null EC9706 cell line was utilized to examine the effect. The results showed that cell cycle G1 phase block and cell proliferation inhibition effects were remarkably enhanced by ECRG1 and ECRG4 co-expression in ESCC cells. It indicated that ECRG1 and ECRG4 might act as co-functional proteins in cell cycle and growth regulation in ESCC.

The cell cycle alteration plays a major role in carcinogenesis. Once the cell cycle regulation balance was broken, it might result in tumorigenesis. Evidence has revealed that many oncogenes and tumor suppressor genes are directly involved in regulation of cell cycle events [28]. The p21 and p16 genes, critical cyclin-dependent kinase inhibitors, were functionally relevant to the regulation of cell cycle G1 phase. In the present research, we observed that ECRG4 and ECRG1 co-expression significantly induced cell cycle G1 phase block through upregulating p21 expression in ESCC cells. However, there was no significant change of p16 expression level in ESCC cells (data not shown). Based on our data, we speculated that ECRG1 and ECRG4 might co-regulate p21 expression to control cell cycle progression in ESCC. It is well known that p21 upregulation is able to block the cell cycle at G1 phase [29,30]. So the p21 overexpression induced by ECRG4 and ECRG1 co-expression could be the possible molecular mechanism for cell cycle G1 phase block and growth suppression in ESCC.

Taken together, we discovered for the first time that ECRG4 directly interacted with ECRG1 to upregulate p21 expression, induce cell cycle G1 phase block and inhibit cancer cells proliferation in ESCC. Our study implied that the interaction of ECRG4 with ECRG1 could be an important therapeutic target for ESCC.

## Competing interests

The authors declare that they have no competing interests.

## Authors' contributions

LL carried out cell cycle analysis, binding affinity assay, co-immunoprecipitation experiment, Western blotting, and gene functional assays. YL and XL analyzed and interpreted the data. SL and YZ supervised experiment. LL and CZ wrote the manuscript. All authors read and approved the final manuscript.

## Abbreviations

ECRG4: esophageal cancer related gene 4; ECRG1: esophageal cancer related gene 1; ESCC: esophageal squamous cell carcinoma; PBS: phosphate-buffered saline

## Pre-publication history

The pre-publication history for this paper can be accessed here:

http://www.biomedcentral.com/1471-2407/11/52/prepub
